# Application of hydrocyclones for continuous cultivation of SP-2/0 cells in perfusion bioreactors: Effect of hydrocyclone operating pressure

**DOI:** 10.1186/1753-6561-5-S8-P65

**Published:** 2011-11-22

**Authors:** Elsayed A Elsayed, Roland Wagner

**Affiliations:** 1 Advanced Chair for Proteomics & Cytomics Research, Zoology Department, Faculty of Science, King Saud University, 11451 Riyadh, Kingdom of Saudi Arabia; 2 Natural & Microbial Products Dept., National Research Centre, Dokki, Cairo, Egypt; 3 Technology Development Department, Rentschler Biotechnology GmbH, D-88471 Laupheim, Germany

## Background

Hydrocyclones (HCs) have been recently extensively evaluated for their application in the separation of mammalian cells in perfusion bioreactors. The high centrifugal force derived within hydrocyclones is the key mechanism for cell separation inside such small-sized simple devices. This is usually accompanied by a very short residence time of the cells inside the separating equipment, usually not more than 0.2 s. Moreover, they have other specific characteristics, which highly recommend their application for the production of pharmaceutical products in perfusion cultivation bioreactors. They are characterized by their high performance, robustness, lack of movable parts, ease of *in situ* sterilization and suitability for cleaning-in-place processes.

## Materials and methods

The hydrocyclone HC 2520, specially designed for cell cultivation, was used for separation of the recombinant mouse lymphoid cell line SP-2/0. It has a 25-mm underflow and 20-mm overflow orifice. A pulsation-free pumphead (Watson Marlow 505L) was fitted to a WM 505DU pump and the perfusion was performed intermittently. Cell were cultured on serum-free ZKT-1 medium supplemented with 5% foetal bovine serum.

## Results

### Effect of hydrocyclone operating pressure on cell growth and cell viability

**Figure 1:**
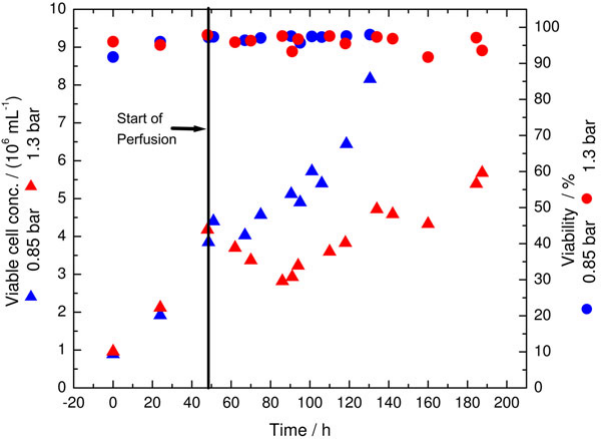
Cell growth and cell viability

The results obtained (Figure 1) showed that cells required an adaptation phase to adapt themselves to the HC operating pressure. Moreover, the length of the adaptation phase depends on the HC pressure. Generally, cells were able to grow with high viability after the operation of hydrocyclone at both tested pressure values (0.85 and 1.30 bar). Maximal cell concentrations of about 8.2 × 10^6^ and 6.0 × 10^6^ mL^-1^ were achieved at an operating pressure of 0.85 and 1.30 bar with a viability range from 92 to 98%, respectively. This corresponds to a 5- and 3.5-fold increase than the batch cultivation, respectively.

### Effect of hydrocyclone operating pressure on separation efficiency and separated cell quality

**Figure 2:**
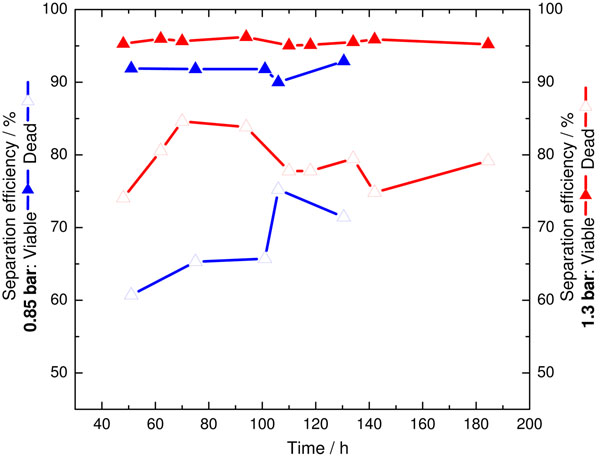
Hydrocyclone separation efficiency

Results (Figure 2) showed that increasing operating pressure from 0.85 to 1.30 bar increased the average total separation efficiency from 89 to 95%. Additionally, results also showed that hydrocyclone preferably separates more viable cells in the underflow, and hence, back to the bioreactor system. Thus, more dead cells are separated in the overflow leaving the bioreactor system, which will improve system viability and product quality.

## Conclusions

- Hydrocyclone can be successfully used for cell separation in continuous mammalian perfusion bioreactors.

- Cells adapt themselves to the shear forces inside the HC without being adversely affected by the pressure.

- Higher separation efficiencies up to 96% can be achieved depending on the operating HC pressure.

- HCs separate preferably more living cells in the underflow, thus more dead cells are leaving the system, which will finally lead to the improvement of system viability and product quality.

